# Association between stunting and neuro-psychological outcomes among children in Burkina Faso, West Africa

**DOI:** 10.1186/s13034-018-0236-1

**Published:** 2018-06-07

**Authors:** Anselme Simeon Sanou, Abdoulaye Hama Diallo, Penny Holding, Victoria Nankabirwa, Ingunn Marie S. Engebretsen, Grace Ndeezi, James K. Tumwine, Nicolas Meda, Thorkild Tylleskär, Esperance Kashala-Abotnes

**Affiliations:** 1 Centre for International Health (CIH), Department of Global Public Health and Primary Health Care, Faculty of Medicine, University of Bergen, Bergen, Norway; 2 Department of Public Health, Centre MURAZ Research Institute, Ministry of Health, Bobo-Dioulasso, Burkina Faso; 3 Department of Public Health, University of Ouagadougou, Ouagadougou, Burkina Faso; 4 Identitea, Nairobi, Kenya; 5 Department of Epidemiology & Biostatistics, School of Public Health, Makerere University, Kampala, Uganda; 6 Centre for Intervention Science in Maternal and Child Health (CISMAC), Department of Global Public Health and Primary Health Care, Faculty of Medicine, University of Bergen, Bergen, Norway; 7 Department of Paediatrics and Child Health, Makerere University, Kampala, Uganda

**Keywords:** Stunting, Nutrition, Neuro-psychological test, KABC-II, CCT-1, TOVA, Children, Burkina Faso, Africa

## Abstract

**Background:**

In Burkina Faso, stunting affects children and is a public health problem. We studied the association between stunting and child’s neuro-psychological outcomes at 6–8 years of age in rural Burkina Faso using the Kaufman Assessment Battery for Children, 2nd edition (KABC-II), the Children’s Category Test 1 (CCT-1) and the Test of Variable of Attention (TOVA).

**Methods:**

We re-enrolled children of a previously community-based Exclusive breastfeeding trial in Burkina Faso. We assessed a total of 532 children aged 6–8 years using KABC-II for memory (Atlantis and Number Recall subtests), spatial abilities (Conceptual Thinking, Face Recognition and Triangle subtests), reasoning (Block Counting subtest), general cognition and CCT-1 for cognitive flexibility. A total 513 children were assessed using the TOVA to measure attention and inhibition. We calculated the Cohen’s d to examine the effect size and conducted a linear regression to examine the association.

**Results:**

The proportion of stunting was 15.6% (83/532). Stunted children performed significantly poorer for memory (Atlantis and Number Recall), spatial abilities (Conceptual Thinking, Face Recognition and Triangle), general cognition and attention with a small effect size compared to non-stunted children. Children who were exposed scored significantly higher errors for cognitive flexibility and inhibition with a small effect size compared to unexposed children. At standardized and unstandardized multivariable regression analysis, stunted children performed significantly poorer for Atlantis (p = 0.001), Number Recall (p = 0.02), Conceptual Thinking (p = 0.01), Triangle (p = 0.001), general cognition (p ≤ 0.0001) and attention (p = 0.04) compared to non-stunted children. Children who were exposed scored significantly higher errors for cognitive flexibility (p = 0.02) and for inhibition (p = 0.02) compared to unexposed children. We adjusted all the results for age, schooling, sex, playing, father education, mother employment and promotion of previous exclusive breastfeeding.

**Conclusion:**

Stunting is associated with poorer neuro-psychological outcomes among children in rural Burkina Faso. Initiatives related to prevention need to be established and advice on nutrition need to be provided.

**Electronic supplementary material:**

The online version of this article (10.1186/s13034-018-0236-1) contains supplementary material, which is available to authorized users.

## Background

Stunting affects more than 165 million children in the world and is highly prevalent from 20 to 35% in sub-Saharan Africa [1, 2]. In Burkina Faso, it is public health problem and varies from 8% for 10–12 years children [3], to 29% for 1–5 years and 8–14 years children [4, 5]. Poor nutrition among children is a major risk factor in several diseases, disabilities, delayed cognitive development in childhood, increased a longer-term risk of chronic disease, reduced income in adulthood and deaths throughout the world [2, 6–9]. It is one of the best overall indicator of children’s well-being and an accurate reflection of social inequalities [10]. Stunting is closely tied to access to services, poverty and causal factors include prenatal and postnatal periods [11–13]. In sub-Saharan Africa, it has several socio-demographic and family factors [14–19].

Many studies in low-income countries have shown that stunting is associated with cognitive outcomes; in different studies, associations were found between stunting and cognitive ability at 5 years, during adolescence and at age 20–22 years [9, 20–22]. Children who experienced stunting in early childhood may have deficiencies related to cognition, school performance and intelligence deficits [23–31]. Also, risk factors of stunting including child’s education, home environment and parental education were found to affect child cognition [32, 33].

More specifically, stunting is associated with verbal comprehension and performance abilities [23], language comprehension, memory [24], vocabulary [24, 27], problem solving and executive function [29], reasoning [31], general cognition [24, 25]. However, the studies showing the effect of stunting on neuro-psychological outcomes used traditional tests administered by human examiner; those tests are non computerized one-on-one tests and some of them are the Bayley mental and motor scales [34], the Weschler Intelligence Scales [35], the Ravens Progressive Matrices [36]. While much is known about poor nutrition association and cognitive outcomes using traditional tests, data from West Africa is scarce and gaps in knowledge still persist in the effect of stunting using computerized neuro-psychological testing.

Children’s neuro-psychological outcomes can be assessed by a variety of neuro-psychological tests. One of the traditional human administered tests is the Kaufman Assessment Battery for Children, Second Edition (KABC-II) [37]. Another human administered test is the first level of the Children’s Category Test (CCT-1) developed to assess cognitive flexibility in children [38]. Both tests were used in the country [39]. The Test of Variables of Attention (TOVA) is a used computerized neuro-psychological (Leark et al. [49]). It measures attention and has been used to explore multiple health and developmental risks in the exploration of attention and was used in Africa [40–42].

Given the gaps of knowledge of the effect of stunting on neuro-psychological outcomes using both traditional and computerized tests in general and in West Africa in particular, we studied the association between stunting and neuro-psychological outcomes using KABC-II, CCT-1 and TOVA among children in Burkina Faso.

## Methods

### Setting, study area, participants and study design

Burkina Faso is a West African country with 46.3% of the population aged 0–14 years, and 70.1% living mainly in rural areas [39, 43, 44]. We re-enrolled children of a previously community-based Exclusive breastfeeding trial in Burkina Faso conducted in 2006 [45]. The sampling and further details of the participants and study site was described [39, 45, 46].

### Outcome measures

The KABC-II is used for children aged 3–18 years and has several subtests [37, 39, 47]. The total raw score of the subtests was used as a measure of general cognition. The KABC-II ‘Atlantis’ and ‘Number Recall’ subtests were used as measures of memory; ‘Conceptual Thinking’, ‘Face Recognition’ and ‘Triangle’ were used as measures of spatial abilities. ‘Block Counting’ was used as a measure of reasoning. The KABC-II subtests ‘Atlantis’, ‘Number Recall’, ‘Conceptual Thinking’, ‘Face Recognition’, ‘Triangle’ and Block Counting’ were considered in the study as they showed good reliability in rural Burkina Faso [39].

The CCT-1 is a test used for children aged 5–8 years and counts the number of errors [38, 39, 48]. In our study, we used the total raw errors as a measure of cognitive flexibility.

The visual TOVA is a computerized test developed to assess attention and inhibition. In our study, we used the TOVA to measure attention and inhibition [41, 49–51]. Attention was measured by the D prime score and inhibition was measured by the error of commission. The D prime score is a response sensitivity score and is interpreted as a measure of accurate performance over time and errors of commission are inappropriate responses to the non-target stimulus [41, 49–51]. Those variables were automatically exported from TOVA on the computer.

In the procedure of administration, TOVA was the first test to be performed, followed by KABC-II and CCT-1. Further details of the administration procedures have been described [39].

### Exposure measure

Stunting at 6–8 years old was the exposure measure. A paediatrician measured anthropometric variables (height, age) at the study site prior to the neuro-psychological testing and according to standard procedures [52]. We defined stunting as below − 2SD of height-for-age. We calibrated the stadiometer according to the instructions of the manual. WHO Anthro was used to classify the children into height for age categories of nutritional status [53].

### Covariates

Socio-economic status, background characteristics’ and clinical history questions were asked prior to neuro-psychological assessments. These include child’s age, schooling, playing with objects at home which was shown to stimulate neuro-psychological outcomes [54], child was beaten in the last 12 months, mother’s age, mother’s education, mother’s employment, mother’s depression (depressed or not depressed) using the Hopkins symptoms depression status [55], father’s education, father’s employment, polygamy, presence of electricity in the compound. It also included history of cerebral malaria and past hospitalizations. Anthropometric measures (weight, height, age) were collected. We defined Underweight as below − 2SD of weight-for-age and thinness as below − 2 SD of BMI-for-age. The promotion of exclusive breastfeeding which was the intervention of the PROMISE EBF trial was retrieved. Further details of the piloting and the field-testing of all the tools have been described [39].

### Statistical analysis

The variance of the population was examined using scores’ distribution (mean, standard deviation, median, minimum and maximum). Covariates’ differences by stunting were tested using student test, Chi square analyses, Fisher exact test. The effect size was examined using Cohen’s d calculation and the association between stunting and the neuro-psychological outcomes was conducted using linear regression. Both unstandardized scores (using raw scores of the neuro-psychological tests) and standardized z-scores (all the raw scores were converted to z-values, mean = 0, SD = 1) were used in the analysis. We adjusted the coefficients for potential confounders [30, 31] and also for the previous intervention. A bivariate analysis was conducted with the covariates (Additional file 1). The statistics tests were declared significant at the 5% level and were two-sided. The analysis was performed using STATA 13. The analysis methodology was previously used [39].

### Ethical considerations

We obtained a written informed consent from all the care-takers and an oral assent from the children. The Institutional Review Board (IRB) of Centre MURAZ has approved the study number 008-2013/CE-CM.

## Results

### Study population

**Fig. 1:**
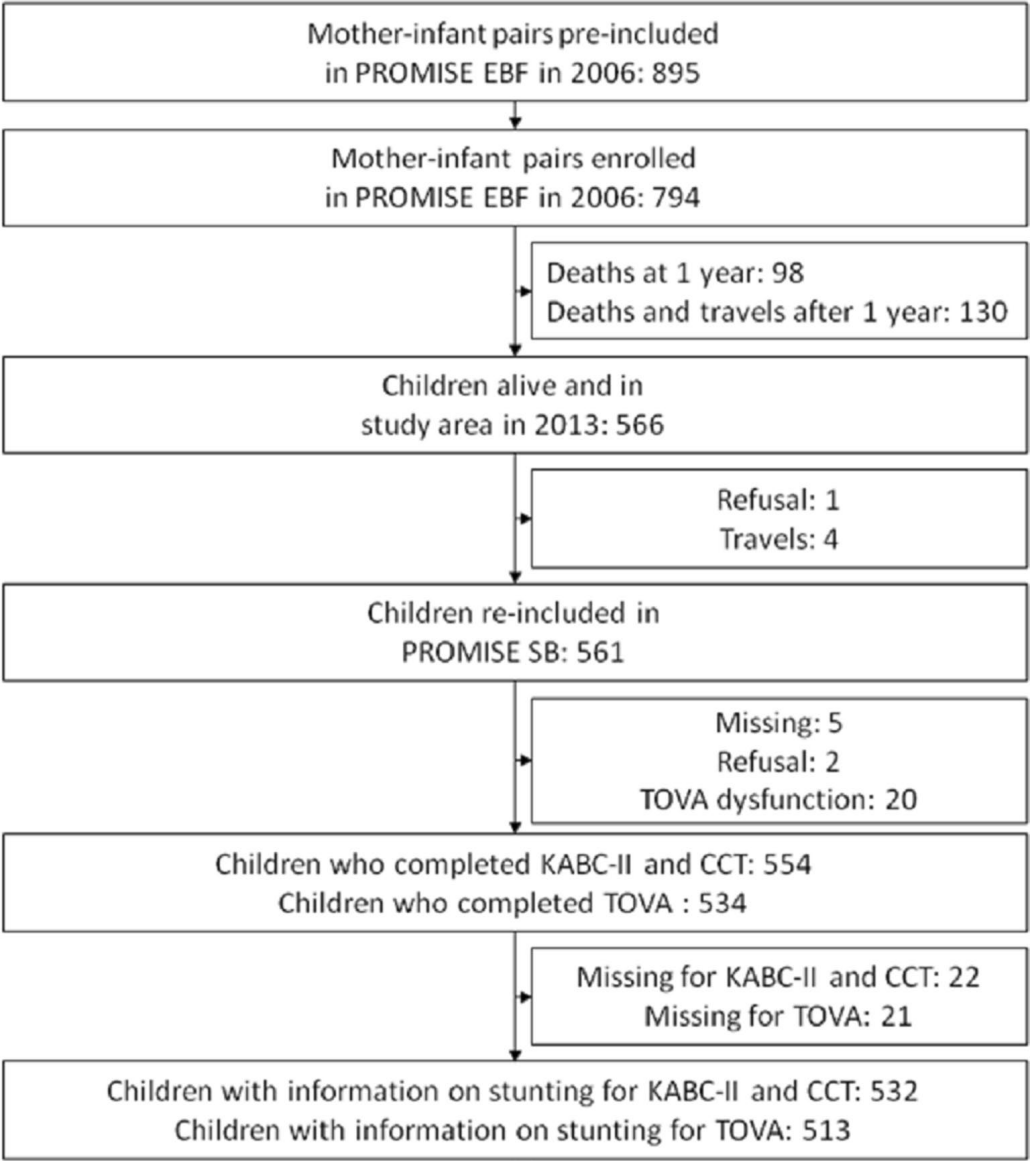
Study profile of children at the PROMISE SB study in Burkina Faso

Of the 794 children enrolled in the previous PROMISE EBF trial, 561 were re-consented for the PROMISE SB follow-up study, 554 children were assessed for neuro-psychological testing, and information on stunting was collected for 532 children (Fig. 1).

Of these, 15.6% (83/532) were stunted, 52.8% (281/532) were boys, and 49.8% (265/532) were at school. Children’s age was ranged from 6.3 to 8.0; the median age (IQR) of the children during assessment was 7.2 (6.9–7.4). Amongst the children, 10.2% (54/531) were underweight, 23.0% (120/522) had history of hospitalization and 47.7% (242/507) played with objects at home.

At the time of assessments, the mean (± SD) age of the mothers was 33.3 (± 6.3 years). Amongst the fathers, 13.4% (68/507) had an employment. Electricity was reported in 77.3% (392/507) (Table 1). Underweight, child’s sex, schooling and mother’s depression status were statistically associated with stunting (p  < 0.05) (Table 1).

**Table 1:** Description of the children who completed KABC-II CCT-1 from PROMISE SB in Burkina Faso

	Total	No stunting	Stunting	P value
	N = 532 N (%)	N = 449 (84.40)	N = 83 (15.60)	
Child age mean ± SD (in years)	7.2 ± 0.4	7.2 ± 0.4	7.2 ± 0.4	0.36
Mothers age mean ± SD (in years)	33.3 ± 6.3	33.4 ± 6.4	33.1 ± 6.0	0.75
Underweight (< − 2 SD in weight-for-age)				≤ 0.0001
No	477 (89.8)	431 (96.0)	46 (56.1)	
Yes	54 (10.2)	18 (4.0)	36 (43.9)	
Thinness (< − 2 SD in BMI-for-age)				0.96
No	512 (96.4)	433 (96.4)	79 (96.3)	
Yes	19 (3.6)	16 (3.6)	3 (3.6)	
Sex				0.01
Girls	251 (47.2)	227 (50.6)	54 (65.1)	
Boys	281 (52.8)	222 (49.4)	29 (34.9)	
Child in school				0.003
Yes	265 (49.8)	236 (52.6)	29 (34.9)	
No	267 (50.2)	213 (47.4)	54 (65.1)	
Child has been hospitalized				0.57
No	402 (77.0)	340 (76.6)	62 (79.5)	
Yes	120 (23.0)	104 (23.4)	16 (20.5)	
Child has history of cerebral malaria				0.11
No	443 (91.1)	380 (92.0)	63 (86.3)	
Yes	43 (8.9)	33 (8.0)	10 (13.7)	
Child plays with object at home				0.77
No	265 (52.3)	222 (52.0)	43 (53.8)	
Yes	242 (47.7)	205 (48.0)	37 (46.2)	
Child was beaten in the last 12 months				0.06
No	483 (95.3)	410 (96.0)	73 (91.3)	
Yes	24 (4.7)	17 (4.0)	7 (8.7)	
Father employed				0.79
Yes	68 (13.4)	58 (13.6)	10 (12.5)	
No	439 (86.6)	369 (86.4)	70 (87.5)	
Father educated				0.19
Yes	153 (30.5)	124 (29.4)	29 (36.7)	
No	348 (69.5)	298 (70.6)	50 (63.3)	
Mother employed				0.11
Yes	26 (5.1)	19 (4.5)	7 (8.8)	
No	481 (94.9)	408 (95.5)	73 (91.2)	
Mothers depression status				0.04
No	263 (52.9)	230 (54.9)	33 (42.3)	
Yes	234 (47.1)	189 (45.1)	45 (57.7)	
Polygamy (father has more than 1 wife)				0.24
No	181 (35.7)	157 (36.8)	24 (30.0)	
Yes	326 (64.3)	270 (63.2)	56 (70.0)	
Electricity in compound				0.15
Yes	392 (77.3)	335 (78.5)	57 (71.3)	
No	115 (22.7)	92 (21.5)	23 (28.7)	
PROMISE EBF intervention				0.10
Control arm	284 (53.4)	233 (51.9)	51 (61.5)	
Intervention arm	248 (46.6)	216 (48.1)	32 (38.5)	

**Fig. 2:**
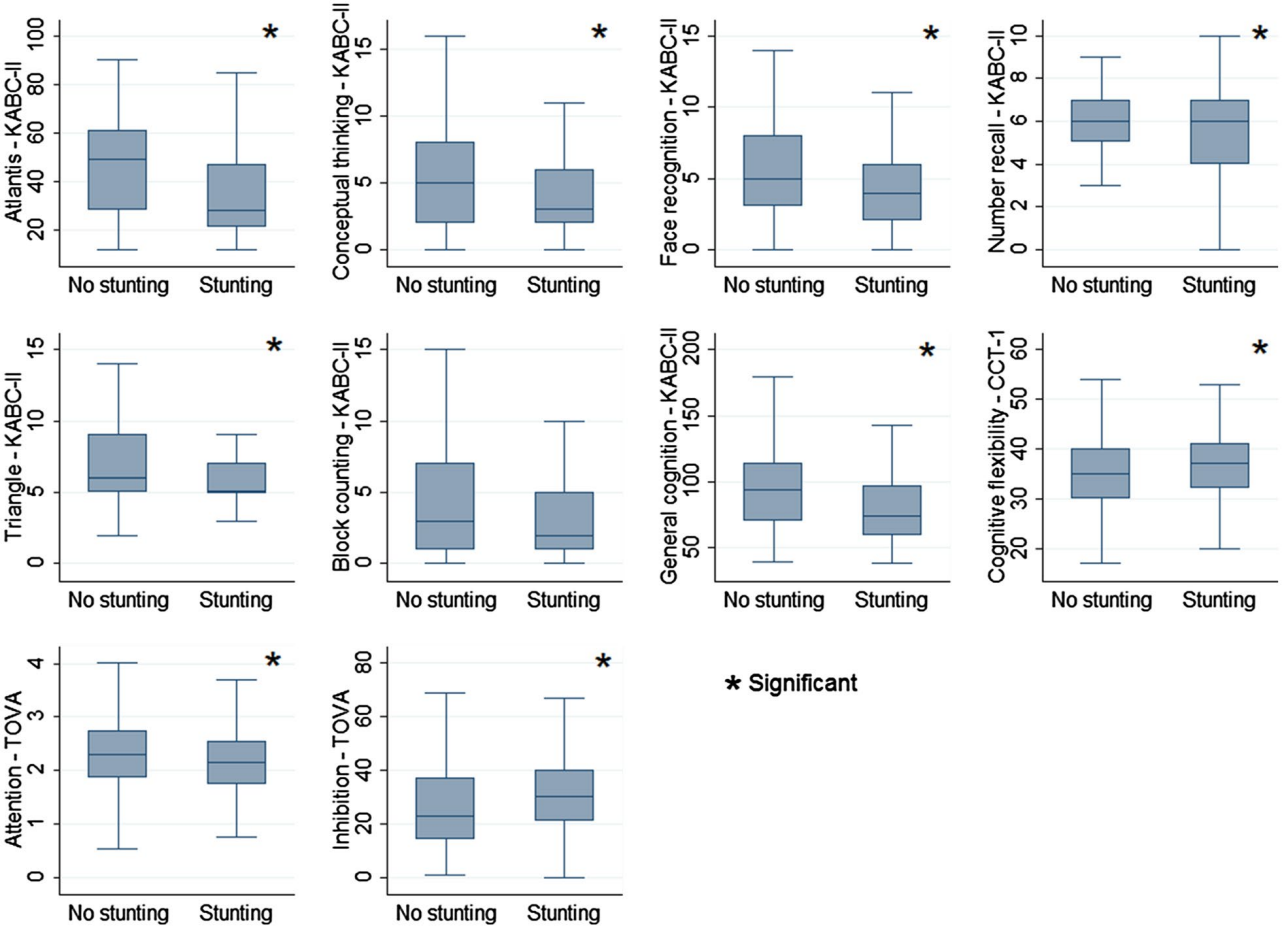
Box-and-whisker plots of neuro-psychological outcomes by stunting from the PROMISE SB study in Burkina Faso

The mean (± SD) of the scores of the tests was 91.6 ± 28.8 for general cognition (KABC-II), 35.6 ± 7.2 for cognitive flexibility (CCT-1), 2.3 ± 0.6 for attention (TOVA) and 27.3 ± 16.5 for inhibition (TOVA) (Fig. 2 and Table 2).

**Table 2:** Neuro-psychological outcomes of children from the PROMISE SB in Burkina Faso

Tests	Unstandardized raw score				Standardized z-score		
	Mean ± SD	Median (IQR)	Min	Max	Median (IQR)	Min	Max
Memory (Atlantis—KABC-II)	43.4 ± 19.3	43 (28–58)	12	90	− 0.02 (− 0.8 to 0.8)	− 1.6	2.4
Visual abilities (Conceptual Thinking —KABC-II)	5.1 ± 3.4	4 (2–8)	0	16	− 0.3 (− 0.9 to 0.8)	− 1.5	3.2
Visual abilities (Face Recognition—KABC-II)	5.0 ± 3.0	4 (3–7)	0	14	− 0.3 (− 0.6 to 0.8)	− 1.6	2.9
Memory (Number Recall—KABC-II)	6.0 ± 1.8	6 (5–7)	0	10	0.05 (− 0.5 to 0.6)	− 3.2	2.2
Spatial abilities (Triangle—KABC-II)	6.7 ± 2.8	6 (5–8)	2	14	− 0.2 (− 0.6 to 0.5)	− 1.7	2.6
Reasoning (Block Counting—KABC-II)	4.1 ± 3.6	3 (1–7)	0	15	− 0.3 (− 0.8 to 0.8)	− 1.1	3.0
General cognition (KABC-II)	91.6 ± 28.8	90 (67–113)	38	179	− 0.04 (− 0.8 to 0.7)	− 1.8	3.0
Cognitive flexibility (CCT-1)	35.6 ± 7.2	35 (31–40)	17	54	− 0.08 (− 0.6 to 0.6)	− 2.6	2.5
Attention (TOVA)	2.3 ± 0.6	2.3 (1.8–2.7)	0.5	4.0	− 0.01 (− 0.7 to 0.6)	− 2.6	2.6
Inhibition (TOVA)	27.3 ± 16.5	24 (15–37)	0	69	− 0.20 (− 0.7 to 0.5)	− 1.6	2.5

*SD* Standard deviation, *IQR* Inter Quartile Range

### Stunting and neuro-psychological outcomes

Stunted children performed significantly poorer for memory (‘Atlantis’ and ‘Number Recall’) and spatial abilities (‘Conceptual Thinking’, ‘Face Recognition’ and ‘Triangle’) tests with a small (between 0.2 and 0.49) effect size difference compared to non-stunted children (Table 3). Stunted children also performed significantly poorer for general cognition (Cohen’s d = 0.48) and attention measure (Cohen’s d = 0.27) with small effect size compared to non-stunted children. Children who were exposed scored significantly higher errors for cognitive flexibility (Cohen’s d = 0.25) and inhibition (Cohen’s d = 0.30) with small effect sizes compared to unexposed (Table 3).

**Table 3:** Effect size and bivariate analysis using linear regression between stunting and outcome measures

	Effect size	Unstandardized	Standardized	P value
	Cohen’s d	Coefficient (95% CI)	Coefficient (95% CI)	
Memory (Atlantis—KABC-II)	0.44^a^	− 8.6 (− 13.1 to − 4.1)	− 0.4 (− 0.6 to − 0.2)	0.0002
Visual abilities (Conceptual Thinking—KABC-II)	0.29^a^	− 0.9 (− 1.8 to − 0.2)	− 0.2 (− 0.5 to − 0.05)	0.01
Visual abilities (Face Recognition—KABC-II)	0.23^a^	− 0.7 (− 1.4 to − 0.01)	− 0.2 (− 0.5 to − 0.002)	0.04
Memory (Number Recall—KABC-II)	0.24^a^	− 0.4 (− 0.9 to − 0.01)	− 0.2 (− 0.5 to − 0.006)	0.04
Spatial abilities (Triangle—KABC-II)	0.42^a^	− 1.2 (− 1.8 to − 0.5)	− 0.4 (− 0.6 to − 0.2)	0.0004
Reasoning (Block Counting—KABC-II)	0.17	− 0.6 (− 1.5 to 0.2)	− 0.2 (− 0.4 to 0.05)	0.1
General cognition (KABC-II)	0.48^a^	− 13.9 (− 20.5 to − 7.2)	− 0.5 (− 0.7 to − 0.2)	≤ 0.0001
Cognitive flexibility (CCT-1)	0.25^a^	1.8 (0.1 to 3.5)	0.3 (0.01 to 0.5)	0.03
Attention (TOVA)	0.27^a^	− 0.2 (− 0.3 to − 0.02)	− 0.3 (− 0.5 to − 0.03)	0.02
Inhibition (TOVA)	0.30^a^	5.0 (1.0 to 8.9)	0.3 (0.06 to 0.5)	0.01

^a^Small effect size from 0.2 to 0.49

At standardized and unstandardized multivariable regression analysis, stunted children performed significantly poorer for memory (p = 0.001 for ‘Atlantis’ and p = 0.02 for ‘Number Recall’) and for Visual abilities (p = 0.01 for ‘Conceptual Thinking’ and p = 0.001 for ‘Triangle’) tests for age, schooling, sex, playing, father education, mother employment and promotion of previous exclusive breastfeeding (Table 4). Stunted children also performed significantly poorer in general cognition (p ≤ 0.0001) and for attention measure (p = 0.04) compared to non-stunted children. The children who were stunted scored significantly higher errors for cognitive flexibility (p = 0.02) and for inhibition (p = 0.02) compared to non-stunted children. We adjusted all the results for age, schooling, sex, playing, father education, mother employment and promotion of previous exclusive breastfeeding (Table 4).

**Table 4:** Linear regression analysis between stunting and KABC-II, CCT-1 and TOVA neuro-psychological outcomes of children from the PROMISE Saving Brains study in Burkina Faso

	Unstandardized	Standardized	P value
	Coefficient^a^ (95% CI)	Coefficient^a^ (95% CI)	
Memory (Atlantis—KABC-II)			
No stunting			
Stunting	− 7.9 (− 12.3 to − 3.4)	− 0.4 (− 0.6 to − 0.2)	0.001
Visual abilities (Conceptual Thinking—KABC-II)			
No stunting			
Stunting	− 1.1 (− 1.9 to − 0.3)	− 0.3 (− 0.6 to − 0.07)	0.01
Visual abilities (Face Recognition—KABC-II)			
No stunting			
Stunting	− 0.7 (− 1.4 to 0.02)	− 0.2 (− 0.5 to 0.01)	0.06
Memory (Number Recall- KABC-II)			
No stunting			
Stunting	− 0.5 (− 1.0 to − 0.07)	− 0.3 (− 0.5 to − 0.04)	0.02
Spatial abilities (Triangle—KABC-II)			
No stunting			
Stunting	− 1.1 (− 1.7 to − 0.5)	− 0.4 (− 0.6 to − 0.2)	0.001
Reasoning (Block Counting—KABC-II)			
No stunting			
Stunting	− 0.7 (− 1.6 to 0.2)	− 0.2 (− 0.4 to 0.04)	0.11
General cognition (KABC-II)			
No stunting			
Stunting	− 13.2 (− 19.7 to − 6.8)	− 0.5 (− 0.6 to − 0.2)	≤ 0.0001
Cognitive flexibility (CCT-1)			
No stunting			
Stunting	1.8 (0.02 to 3.5)	0.2 (0.003 to 0.5)	0.04
Attention (TOVA)			
No stunting			
Stunting	− 0.2 (− 0.3 to − 0.02)	− 0.2 (− 0.5 to − 0.03)	0.02
Inhibition (TOVA)			
No stunting			
Stunting	4.6 (0.5 to 8.8)	0.3 (0.03 to 0.5)	0.02

^a^Adjusted for age, sex, schooling, playing, father education, mother employment and EBF (N = 499 for KABC-II & CCT-1 and N = 481 for TOVA)

## Discussion

In our study, we found that stunting was associated with poorer neuro-psychological outcomes for memory (‘Atlantis’—KABC-II and ‘Number Recall’—KABC-II), spatial ability (‘Conceptual Thinking’—KABC-II and ‘Triangle’—KABC-II), general cognition (KABC-II), cognitive flexibility (CCT-1), attention (TOVA) and inhibition (TOVA) among aged 6–8 years old children in rural Burkina Faso.

The study was carried out in an African rural context where stunting is prevalent and is a public health problem. Three main pathways explain how stunting may affect cognitive outcomes in children: first, a lack of nutrients can damage the brain; second, malnourished children lack the energy to interact with their peers affecting their learning; third, smaller children who appear younger than their age may receive less stimulation from adult expectations than larger children [56]. Our findings compares well with other studies which found that stunted children performed poorly and had much lower scores than adequately nourished children on cognitive tests [29, 30, 57, 58]. In addition, stunted children have a disadvantage regarding reasoning skills needed for their education in early grades [31]. A review highlighted that childhood under nutrition was associated with concurrent and longer term deficits in cognition [59].

In our results, we found several socio-demographic and family factors including sex, education, maternal depression, which were associated with stunting. These results were found in other studies [14–19]. Sex difference varies in stunting; while some studies demonstrated higher levels of stunted boys [60–62], others demonstrated higher levels of stunted girls [63, 64]. Our study found a larger percentage of stunted girls than boys. The reason could be the increased access to food due to the cultural preference of boys at birth [65, 66]. The association between higher education and low stunting could be explained by the fact that educated people are more likely to take decisions which will improve their nutrition [67]. Regarding the effect of maternal depression on stunting, it could be explained by the fact it is associated with deficient child’s psychological, emotional and physical stimulation, a reduced interest in infant caring activities, and unhealthy lifestyles [68, 69]. Our results also found less stunting children in the exclusive breastfeeding group compared to the control group. This could be explained by the fact that liquids different from breast-milk increases the risk of disease, which may result in micronutrient deficiencies and growth retardation [70]. However, exclusive breastfeeding was not associated with stunting. Different studies did not find any effect of exclusive breastfeeding in growth [70–73].

There are several strengths in our study. Firstly, there is a small selection bias risk; the participants were included in a previous community-based trial [45, 74]. Secondly, height was measured according to standardized procedures and with a calibrated stadiometer. Thirdly, all the measurements were based on widely used of standardized measures of neuro-psychological outcomes for children in Africa [41, 47, 75–78]. Also, only trained blinded to stunting psychologists assessed the children [39]. Finally, we adjusted for potential confounders in the analysis.

However, there are some limitations in the study. The instruments were not normed and validated in our setting. This may have affected the outcomes of the children in general. The assumption of cultural inappropriateness reducing the outcomes of neuro-psychological tests was described in several studies [39, 79, 80].

We still consider the manuscript to be relevant as it shows an association between stunting and poor neuro-psychological outcomes in Burkina Faso. The study raises the need to highlight awareness of risks of poor nutrition on children’s neuro-psychological outcomes specially memory, spatial abilities, general cognition, cognitive flexibility, attention and inhibition. Several multisector interventions including health, breastfeeding promotion, complementary feeding, education, agriculture, women empowerment, infrastructure, water, sanitation and hygiene were successfully used to improve child nutrition in low-income countries [81–83]. Joint prevention strategies may then have important roles in reducing poor nutrition and improving neuro-psychological outcomes.

## Conclusion

Stunting is associated with poorer neuro-psychological outcomes among children in rural Burkina Faso. Initiatives related to prevention need to be established and advice on nutrition need to be provided.

## Additional file

**Additional file 1.** Crude coefficient from linear regression between covariates and the neuro-psychological outcomes.
