# Supplementary material for: Association between stunting and neuro-psychological outcomes among children in Burkina Faso, West Africa
Source: Child Adolesc Psychiatry Ment Health. 2018 Jun 7;12:30. doi: 10.1186/s13034-018-0236-1 (PMC5992697; doi:10.1186/s13034-018-0236-1)
Supplement: Supplementary file 1 — Additional file 1. Crude coefficient from linear regression between covariates and the neuro-psychological outcomes. [file 13034_2018_236_MOESM1_ESM.docx]

**Additional file 1:** Crude coefficient from linear regression between covariates and the neuro-psychological outcomes

|  | General cognition | Cognitive flexibility | Attention | Inhibition |
| --- | --- | --- | --- | --- |
| Age, N  Crude, 95% CI  p-value | 532  9.8 (2.9 – 16.6)  0.005 | 532  -1.1 (-2.8 - 0.6)  0.2 | 513  0.3 (0.1 – 0.5)  0.001 | 513  -3.8 (-7.8 – 0.3)  0.06 |
| Sex, N  Crude, 95% CI  p-value | 532  5.1 (0.2 – 10.0)  0.04 | 532  -0.9 (-2.2 – 0.2)  0.1 | 513  -0.1 (-0.2 – 0.01)  0.07 | 513  1.8 (-1.0 – 4.7)  0.2 |
| Child in school, N  Crude, 95% CI  p-value | 532  17.9 (13.2 – 22.6)  0.0001 | 532  -1.3 (-2.5 - -0.1)  0.03 | 513  0.3 (0.2 – 0.4)  0.0001 | 513  -4.5 (-7.4 - -1.7)  0.002 |
| Child plays with object  Crude, 95% CI  p-value | 507  -3.0 (-8.1 – 2.0)  0.2 | 507  -0.5 (-1.8 – 0.7)  0.3 | 488  -0.004 (-0.1 – 0.1)  0.9 | 488  -2.5 (-5.5 – 0.4)  0.08 |
| Father educated, N  Crude, 95% CI  p-value | 501  10.5 (5.1 – 15.9)  0.0001 | 501  -1.2 (-2.6-0.2)  0.08 | 483  0.09 (-0.03 – 0.2)  0.1 | 483  0.06 (-3.1 – 3.3)  0.9 |
| Mother’s employment, N  Crude, 95% CI  p-value | 507  12.6 (1.2 – 24.0)  0.03 | 507  -3.7 (-6.6- -0.8)  0.01 | 488  0.1 (-0.1 – 0.4)  0.3 | 488  1.2 (-5.3 – 7.8)  0.7 |
| PROMISE EBF intervention, N  Crude, 95% CI  p-value | 532  -2.9 (-7.8 – 2.0)  0.2 | 532  1.3 (0.04-2.5)  0.04 | 513  -0.07 (-0.2 – 0.04)  0.2 | 513  0.8 (-2.0 – 3.7)  0.5 |
